# VSNL1 Promotes Gastric Cancer Cell Proliferation and Migration by Regulating P2X3/P2Y2 Receptors and Is a Clinical Indicator of Poor Prognosis in Gastric Cancer Patients

**DOI:** 10.1155/2020/7241942

**Published:** 2020-12-09

**Authors:** Qiao-Qiong Dai, Yuan-Yu Wang, Yu-peng Jiang, Li Li, Hui-Ju Wang

**Affiliations:** ^1^Department of Anus & Intestine Surgery, Zhejiang Provincial People's Hospital, People's Hospital of Hangzhou Medical College, Hangzhou 310014, China; ^2^Departments of Gastrointestinal and Pancreatic Surgery, Zhejiang Provincial People's Hospital, People's Hospital of Hangzhou Medical College, Hangzhou 310014, China; ^3^Key Laboratory of Gastroenterology of Zhejiang Province, Hangzhou 310014, China; ^4^Departments of Gastrointestinal Surgery, Tongxiang First People's Hospital, Jiaxing 314500, China; ^5^Clinical Research Institute, Zhejiang Provincial People's Hospital, People's Hospital of Hangzhou Medical College, Hangzhou, 310014 Zhejiang, China

## Abstract

**Purpose:**

The aim of this study was to investigate the role of Visinin Like 1 (VSNL1) in the proliferation and migration of gastric cancer (GC) cells as well as its clinical prognostic significance.

**Methods:**

To this end, we evaluated VSNL1 expression in GC tissues and cell lines by real-time PCR and immunohistochemistry. To further explore the effects of VSNL1, a lentiviral vector expressing a short hairpin RNA (shRNA) against VSNL1 was constructed and transduced into the GC cell lines BGC-823 and SGC-7901. The interference efficiency of VSNL1-shRNA was determined by western blot. The effects of VSNL1 on the migration and invasion of GC cells as well as the expression of P2X3/P2Y2 were explored using MTS, colony formation, migration, and western blot assays.

**Results:**

VSNL1 mRNA and protein levels were increased in GC tissues and cell lines. Furthermore, VSNL1 expression was positively correlated with Lauren's classification, lymph node metastasis, distant metastasis, TNM stage, and prognosis. VSNL1 expression was inversely correlated with the 5-year survival rate of GC patients. VSNL1 expression was markedly reduced in cells transduced with lentivirus expressing shRNA against VSNL1, and inhibiting VSNL1 expression significantly suppressed cell growth, migration, and colony formation and reduced the expression of P2X3/P2Y2.

**Conclusion:**

VSNL1 may promote the proliferation and migration of GC cells by regulating P2X3 and P2Y2 expression. VSNL1 plays important roles in GC development and metastasis and may be correlated with patient prognosis.

## 1. Introduction

Visinin Like 1 (VSNL1; also known as VILIP-1) is a member of the neuronal calcium sensor protein family and regulates calcium-dependent cellular signaling and signal transduction by modulating adenylate cyclase [1]. The majority of current studies on VSNL1 have focused on Alzheimer's disease and acute encephalopathy [2, 3]; however, VSNL1 is overexpressed in squamous cell carcinoma, neuroblastoma, non-small-cell lung cancer, colorectal cancer, and other tumor types, where it is involved in tumor invasion and metastasis [4–7].

Recent studies have shown that P2X/P2Y signaling plays important roles in inflammatory responses, metabolism, and cancer [8, 9]. Interactions between the amino terminus of VSNL1 and the carboxyl terminus of the P2X3 receptor are critical for P2X3 surface expression and functional enhancement. VSNL1 overexpression increases the expression of P2X3 receptors and enhances the excitability of naïve rat dorsal root ganglion (DRG) neurons [10]. Additionally, binding of the P2Y2 receptor to extracellular ATP promotes the proliferation and migration of cancer cells in nude mice [11, 12].

Few studies have examined VSNL1 expression and function in gastric cancer (GC). Therefore, in this study, we examined VSNL1 expression in GC, correlations between its expression and clinicopathological factors, relationships between VSNL1 and P2X/P2Y receptors, the prognostic value of VSNL1 in GC, and its potential functions in GC progression.

## 2. Materials and Methods

### 2.1. Bioinformatics Analyses Using Oncomine Databases

VSNL1 mRNA expression in GC and normal tissues was compared using Oncomine databases. Analyses were performed online (https://www.oncomine.org) with the following filtering conditions: gene: VSNL1; analysis type: cancer vs. normal analysis; cancer type: GC; data type: mRNA; *p* < 0.05; fold change > 2; and gene rank: top 10%. In total, five analyses were selected, but one was excluded due to insufficient sample size (*n* < 30). Therefore, the remaining four analyses were used to determine VSNL1 expression in tissues.

### 2.2. Tumor Samples

All GC and nontumor mucosa samples were collected from surgical resections performed at the Department of Surgery at Zhejiang Provincial People's Hospital from January 1998 to January 2004. In total, 436 GC patients, who were diagnosed by surgeons and pathologists, were included; among them, 311 (71.33%) were male and 125 (29.77%) were female, and their age ranged from 17 to 91 years. As negative controls, 92 nontumor mucosa samples (distance from tumor margins > 5 cm) were collected from gastrectomy. All cancer patients received routine chemotherapy after surgery and no radiation. The mean follow-up time was 60 months by the end of December 2008. Survival time was measured from the date of surgery to the follow-up deadline or date of death, which mainly resulted from carcinoma recurrence or metastasis. The Review Board of our Hospital Ethics Committee approved the study, and informed consent was obtained from each participant before data collection. All experiments were performed in accordance with relevant guidelines and regulations of our Hospital Ethics Committee.

### 2.3. Cell Culture

The human GC cell lines MKN-45, SGC-7901, BGC-823, and AGS and the nonmalignant gastric epithelial cell line GES-1 were obtained from the Key Laboratory of Gastroenterology of Zhejiang Province (Hangzhou, China) and were cultured in RPMI1640 containing 10% fetal bovine serum (FBS), 50 U/ml penicillin, and 50 *μ*g/ml streptomycin. Cells were maintained at 37°C under an atmosphere of 5% CO_2_.

### 2.4. Immunohistochemistry (IHC) Staining and Evaluation

VSNL1 IHC was performed with mouse anti-VSNL1 (1 : 500; Santa Cruz Biotechnology, Dallas, TX, USA) using protocols previously described in detail [13]. Stained sections were reviewed and evaluated by two independent pathologists. All slides were observed under a Nikon light microscope (Nikon, Tokyo, Japan), and representative images were captured. Percentages of positively stained cells were scored as follows [14]: 0 (≤5%), 1 (6%–25%), 2 (26%–50%), and 3 (>51%). Intensity was scored as 0 (no staining), 1 (weak staining), 2 (moderate staining), and 3 (strong staining). Finally, the percentage score was multiplied by the staining intensity score. The threshold for VSNL1 was based on heterogeneity using the log-rank test with respect to overall survival (OS). A staining index score of ≥4 was defined as high VSNL1 expression and <4 as low.

### 2.5. Construction and Transduction of the VSNL1 shRNA Lentiviral Vector

According to the VSNL1 mRNA sequence, three lentiviral shRNA constructs targeting VNSL1 (VNSL1 shRNA-1: 5′-CCCTTCCATTGTATTACTT-3′, VNSL1 shRNA-2: 5′-ATGTGAAGTTCTTTCCTTA-3′, and VNSL1 shRNA-3: 5′-ATGAACTCAAGCAGTGGTA-3′) as well as an shRNA noncoding (NC) control lentivirus (TTCTCCGAACGTGTCAT) were purchased from GeneChem Gene (Shanghai GeneChem, Shanghai, China). The three shRNAs were used as a cocktail directed against the coding region of VSNL1. Then, BGC-823 and SGC-7901 cells were transduced with VSNL1 shRNA-1, shRNA-2, and shRNA-3 in 6-well plates (2 × 10^5^ cells/well) to inhibit VSNL1 expression; shRNA-NC was transduced as a negative control. Cells were cultured if GFP expression was observed under the microscope in more than 50% of cells 3 d after infection.

### 2.6. In Vitro Cell Migration and Invasion Assays

For migration assays, infected GC cells (BGC-823 and SGC-7901; 1 × 10^5^) were plated in triplicate in the top chamber of Transwell plates (Millicell Hanging Cell Culture Inserts, PIEP12R48, Millipore Corporation, Burlington, MA, USA) with a membrane containing 8 *μ*m diameter pores in 250 *μ*l of serum-free RPMI1640. The inserts were then placed into the bottom chamber wells of a 24-well plate containing RPMI1640 with 20% FBS as a chemoattractant. After 24 h incubation, cells remaining on the insert top were removed with a cotton swab, while cells on the lower surface of the membrane were fixed in 100% methanol for 15 min, followed by staining with Giemsa solution. Cell numbers in five random fields (200x) were counted for each chamber to calculate average values.

For invasion assays, infected cells (1 × 10^5^) were plated in the top chamber of Matrigel-coated membranes (QCM ECMatrix Cell Invasion Assay, Millipore Corporation), whereas the bottom chambers were filled with conditioned medium. After 24 h incubation, migrated cells (lower side of the membrane) were counted as described above.

### 2.7. Colony Formation Assay

Three days after infecting cells, cells in the logarithmic phase were digested with trypsin, resuspended, counted, and seeded into 6-well plates at 200 cells per well in triplicate. The cells were incubated for 14 d to form colonies. Then, cells were washed with PBS, fixed with absolute ethanol for 15 min, stained with crystal violet for 10 min, washed three times with H_2_O, air dried, and then photographed with a digital camera. The number of colonies (>50 cells) was counted under a fluorescence microscope (MicroPublisher 3.3 RTV; Olympus, Tokyo, Japan). All assays were performed in triplicate.

### 2.8. Cell Proliferation Assay

The effect of VSNL1 on the proliferation of GC cells was evaluated by MTS assays. BGC-823 and SGC-7901 cells were seeded into 96-well plates at a density of 2.0 × 10^3^ cells/well in quintuplicate and allowed to adhere overnight. Then, 50 *μ*l PMS was added to 1 ml MTS, and then, 20 *μ*l was added to each well. Finally, OD values were read at 490 nm after incubating with MTS for 4 h. Cell growth was observed continuously for 3 d, and cell growth curves were drawn.

### 2.9. Protein Extraction and Western Blot Analysis

Total protein was extracted using RIPA lysis buffer (Beyotime, China) on ice for 30 min, 12000 rpm centrifugation 10 min; supernatant was collected, and protein content was determined by BCA (Pierce, Rockford, IL, USA), and 40 *μ*g protein of each sample was applied to 12% SDSPAGE. To analyze the expression level of VSNL1 and *β*-actin, separated proteins were blotted on a PVDF membrane. The membrane was blocked with 5% milk powder in TBST (25 mM Tris, 150 mM NaCl, pH 7.5, 1% Tween 20) for 1 hour at RT and afterwards incubated with the antibodies (VSNL1: 1 : 100; *β*-actin: 1 : 5000) at 4°C overnight. After washing three times with TBST, secondary antibodies (VSNL1 : rabbit anti-human: 1 : 4000, *β*-actin : mouse anti-human: 1 : 4000) were applied for 2 hours at RT.

### 2.10. Western Blot Analysis

Total cellular protein was extracted as described previously [13]. Protein concentrations were quantified with a BCA protein assay kit (Pierce, Waltham, MA, USA). Western blotting was performed with established procedures. A rabbit polyclonal anti-P2X3 antibody (Abcam, Cambridge, UK), mouse polyclonal anti-P2Y2 antibody (Abcam), and anti-GAPDH antibody (Cell Signaling Technology, Danvers, MA, USA) were used as primary antibodies. GAPDH served as the loading control.

### 2.11. Statistical Analysis

All statistical analyses were performed using SPSS 19.0 software (IBM, Armonk, NY, USA). Student's *t*-test and *χ*^2^ or Fisher's exact test were employed for continuous and categorical data, respectively. Survival curves were estimated using the Kaplan-Meier method, and the log-rank test was used to calculate differences between the curves. Multivariate analysis using the Cox proportional hazards regression model was performed to assess the prognostic values of gene expression. Values were accepted as significant when *p* was <0.05 (∗), <0.01 (∗∗), or <0.001 (∗∗∗). All error bars represent standard deviations.

## 3. Results

### 3.1. VSNL1 Expression in GC Tissues and Cell Lines

**Figure 1:**
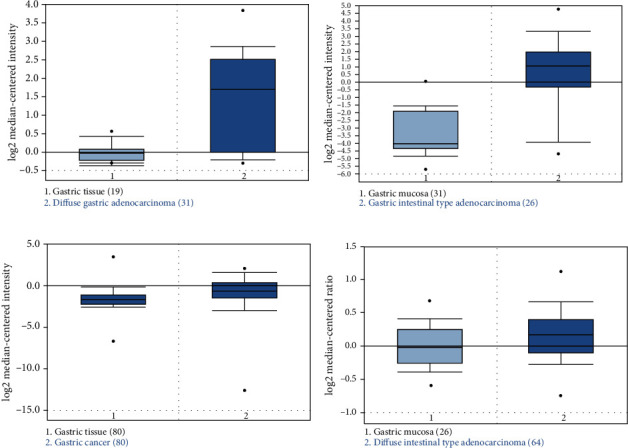
VSNL1 expression in gastric cancer patients. (a–d) The relative level of VSNL1 mRNA was significantly higher in gastric cancer than normal tissues. All data collection and statistical analyses were performed on the Oncomine platform (https://www.oncomine.org).

We analyzed the Oncomine databases to compare VSNL1 mRNA expression in GC versus normal tissues. There were four available datasets regarding VSNL1 in GC. We found that VSNL1 mRNA expression was significantly higher in gastric cancer tissues, including those of different differentiation types, compared with normal tissues (Figures 1(a)–1(d), all *p* < 0.05). A synthetic comparison across these four analyses further confirmed VSNL1 overexpression in GC tissues (*p* < 0.05).

**Figure 2:**
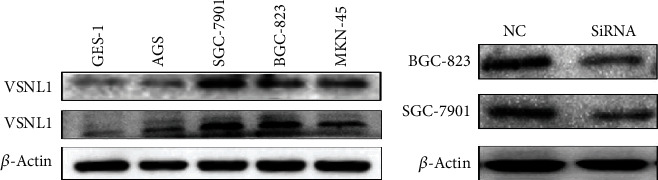
VSNL1 expression in GC cell lines. We repeated each experiment three times. (a) VSNL1 was highly expressed especially in SGC-7901 and BGC-823. (b) The expression of VSNL1 was significantly downregulated after transfection.

VSNL1 expression in the human GC cell lines MKN-45, SGC-7901, BGC-823, and AGS and the nonmalignant gastric epithelial cell line GES-1 was detected by western blot, and the results showed that VSNL1 was highly overexpressed in GC cells, especially SGC-7901 and BGC-823 (Figure 2(a)). VSNL1 expression was significantly downregulated after transducing VSNL1 shRNA (Figure 2(b)).

### 3.2. VSNL1 Expression in 436 GC Tissues

**Figure 3:**
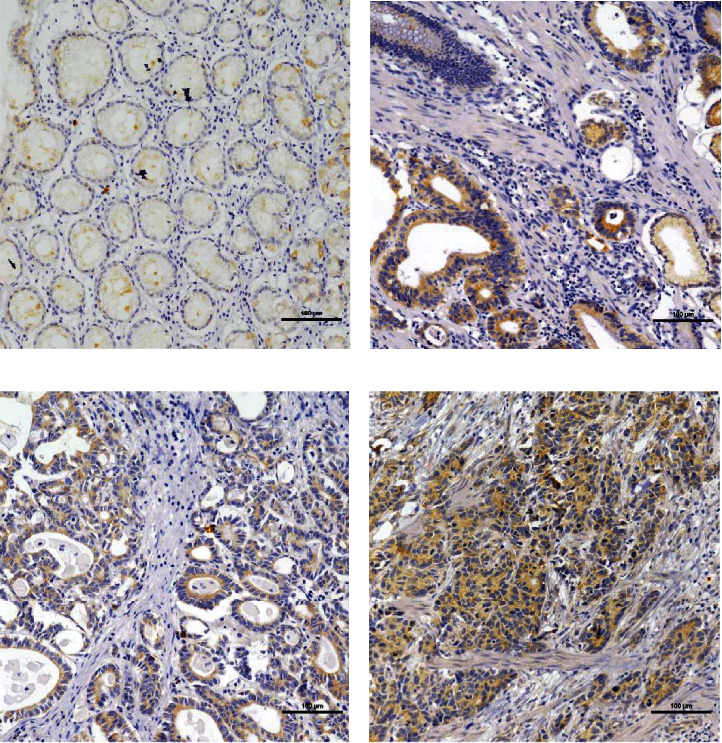
VSNL1 expression determined in gastric cancer lesions and noncancerous tissues by IHC; magnification: ×200. (a) VSNL1 was weakly expressed in noncancerous tissues. (b, c) VSNL1 was mainly localized in the cytoplasm of cancer cells; VSNL1 was highly expressed in moderately differentiated adenocarcinoma. (d) VSNL1 was highly expressed in poorly differentiated adenocarcinoma.

Among the 92 control human nontumor mucosa samples, VSNL1 protein was weakly expressed in 16 (17.39%) samples. In contrast, VSNL1 was detected in 201 (46.10%) cases of human GC, among which 172 (39.45%) cases showed high VSNL1 expression. VSNL1 was primarily localized in the cytoplasm and nuclei of cancer cells (Figure 3).

### 3.3. Associations between VSNL1 Expression and Clinicopathological Features and Prognoses

VSNL1 expression was significantly correlated with age, tumor size, depth of invasion, Lauren's classification, lymph node and distant metastases, regional lymph node stage, and TNM stage (all *p* < 0.05). VSNL1 expression was not significantly correlated with sex, tumor location, differentiation, or histological classification (*p* > 0.05; Table 1).

**Figure 4:**
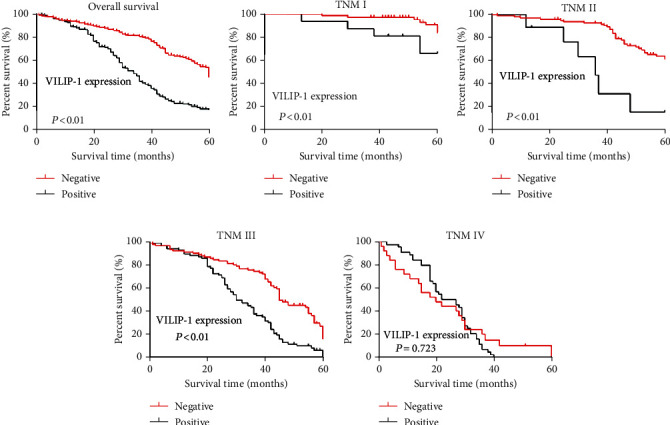
Kaplan-Meier curves with univariate analyses (log-rank) for patients with low VSNL1 expression versus high VSNL1 expression. (a) Cumulative 5-year survival rates for patients with high VSNL1 expression were significantly lower than in patients with low VSNL1 expression. (b–d) According to the TNM stage, in stages I, II, and III, the patients with high expression of VSNL1 had a significantly lower 5-year survival rate than those with high expression (*p* < 0.05). (e) In stage IV, the expression of VSNL1 was not correlated with the 5-year survival rate (*p* > 0.05).

The cumulative 5-year survival rate for patients with high VSNL1 expression was significantly lower than that of the low VSNL1 expression group (Figure 4(a)). We further analyzed correlations between VSNL1 expression and prognosis by Kaplan-Meier curves according to the TNM stage. For stages I–III, patients with higher VSNL1 expression had significantly lower 5-year survival rates than those with lower expression (*p* < 0.05). For stage IV, VSNL1 expression was not correlated with 5-year survival rates (*p* > 0.05; Figures 4(b)–4(e)).

### 3.4. The Effect of VSNL1 on the Proliferation and Migration of GC Cells *In Vitro*

**Figure 5:**
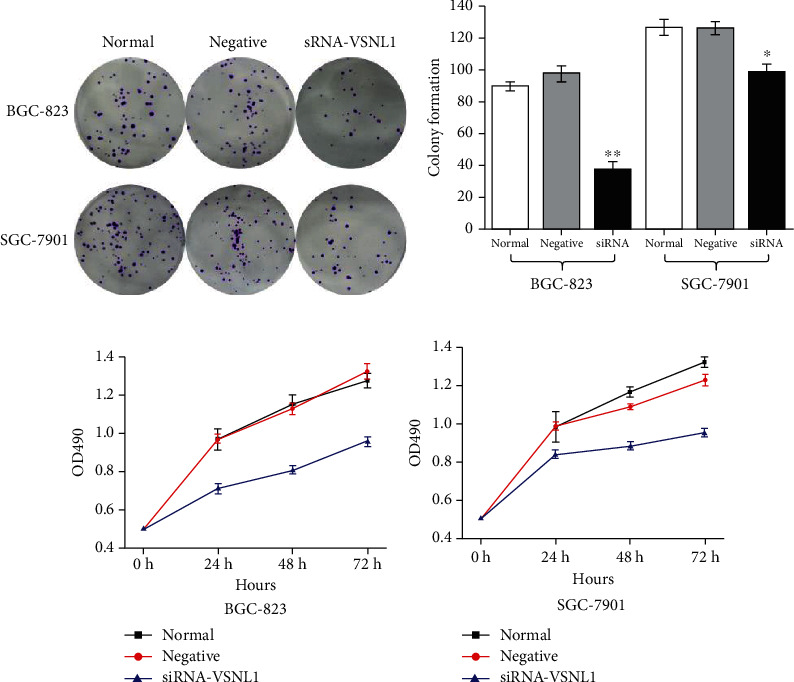
The effect of VSNL1 on the proliferation of GC cells in vitro. We repeated each experiment three times. (a, b) Colony formation capacity was reduced following VSNL1 silencing (*p* < 0.05). (c, d) Downregulation of VSNL1 expression inhibited the proliferative ability of BGC-823 and SGC-7901 cells compared with negative control and normal control cells (*p* < 0.05).

**Figure 6:**
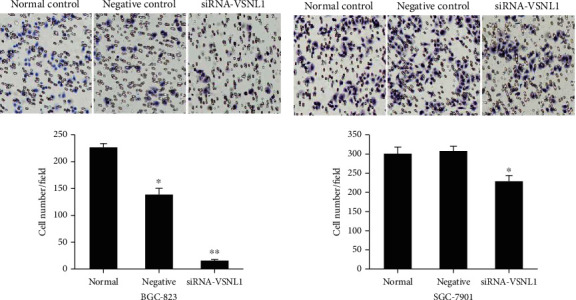
The effect of VSNL1 on the migration and invasion of GC cells in vitro. (a, b) Migration was inhibited significantly when VSNL1 was depleted in BGC-823 cells; migration was inhibited slightly when VSNL1 was depleted in SGC-7901 cells.

Colony formation capacity was reduced following VSNL1 silencing (*p* < 0.05; Figures 5(a) and 5(b)). Similarly, silencing VSNL1 inhibited the proliferation of BGC-823 and SGC-7901 cells compared with negative control and normal control cells (*p* < 0.05; Figures 5(c) and 5(d)). VSNL1 also increased the migration and invasion of GC cells. Finally, we performed Transwell assays to evaluate the role of VSNL1 in GC cell migration. Migration was inhibited significantly when VSNL1 was depleted in BGC-823 cells; migration was inhibited slightly when VSNL1 was depleted in SGC-7901 cells (Figure 6).

### 3.5. Decreased P2X3/P2Y2 Expression in GC Cells after Transducing VSNL1 shRNA

**Figure 7:**
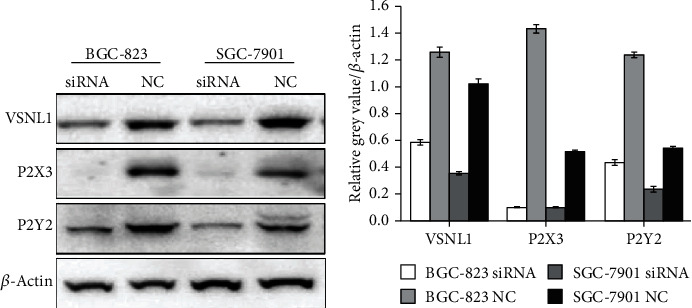
The effect of VSNL1 on the expression of P2X3/P2Y2. We repeated each experiment three times. Substantially decreased P2X3/P2Y2 expression was found in BGC-823 and SGC-7901 cells 72 h after infection with VSNL1 shRNA2.

Substantially decreased P2X3/P2Y2 expression was found in BGC-823 and SGC-7901 cells 72 h after infection with VSNL1 shRNA2 (Figure 7).

## 4. Discussion

Stomach cancer is the second and third leading cancer type in men and women, respectively, in China. However, stomach cancer is the second leading cause of cancer death for both sexes, and the main causes of GC-related death are invasion and metastasis [15]. Tumor cell invasion and migration is a very complicated and continuous process that is regulated by sustained proliferative signaling, evading growth suppressors, resisting cell death, and enabling replicative immortality [16].

VSNL1 is a member of the neuronal EF-hand calcium sensor protein family and plays a role in regulating tumor cell invasion and migration. VSNL1 decreases cellular adhesion and the migration/invasiveness of highly invasive mouse squamous cell carcinoma cells [4]. VSNL1 was significantly highly expressed in highly invasive neuroblastoma cells [5] and in gastric noncardiac adenocarcinomas by the gene expression profiles of GSE29272 [17]. Our study showed that VSNL1 was more highly expressed in GC cells and tissue and that VSNL1 expression was significantly correlated with lymph node and distant metastases and TNM stage. VSNL1 was also found to be overexpressed in neuroblastoma specimens from patients with distant metastases [5]. VSNL1 mRNA was also significantly highly expressed in colorectal cancer tissue with lymph node metastasis. Furthermore, stage III patients with high VSNL1 expression had significantly poorer prognoses [7]. The Tumor-Node-Metastasis (TNM) stage is an important factor for determining GC outcomes. Therefore, we further analyzed the effect of VSNL1 on prognosis according to the TNM stage. In stages I–III, patients with higher VSNL1 expression had significantly lower 5-year survival rates than those with high expression. VSNL1 is involved in epithelial-mesenchymal transition (EMT) in squamous cell carcinoma [18], and VSNL1 might regulate the aggressiveness of murine dermal squamous cell carcinoma cells [19]. Our results showed that silencing VSNL1 inhibited the proliferation, migration, and invasion of GC cells.

Expressing purinergic receptors is an additive advantage for tumor cells because specific subtypes support cancer growth and invasiveness, while decreasing the efficacy of immune surveillance [20]. P2X3 is overexpressed in hepatocellular carcinoma, where high P2X3 expression is associated with poor recurrence-free survival. Moreover, P2X3 antagonism attenuated nucleotide-induced proliferation and cell cycle progression [21]. P2Y2 shows higher expression in the highly metastatic breast cancer cell line MDA-MB-231, where it promotes breast cancer growth and metastasis [22, 23]. P2Y2 levels are higher in hepatocellular carcinoma and in hepatocellular carcinoma cell lines. Finally, binding of the P2Y2 receptor with extracellular ATP promotes the proliferation and migration of cancer cells in nude mice [11, 12].

VSNL1 is an important modulator of tumor biology that regulates the expression of adenylyl cyclase in a cAMP-dependent manner [18, 19]. VSNL1 may also directly bind to P2X/P2Y receptors to regulate their activity [10]. Our study found that after 72 h infection with VSNL1 shRNA2, the expression of P2X3 and P2Y2 decreased substantially.

VSNL1 promotes the proliferation, migration, and invasion of GC cells by regulating the expression of P2X3 and P2Y2 receptor. VSNL1 plays important roles in GC development and metastasis and may be associated with poor prognoses.

## Figures and Tables

**Table 1:** Relationship of VILIP-1 expression with pathological parameters of GC.

Clinical parameters	VILIP-1			
	Low	High	*t*/*χ*^2^	*p*
Age (yrs)	57.61 ± 11.38	61.62 ± 12.06	-3.781	<0.01
Gender			0.081	0.776
Male	187 (60.1%)	124 (39.9%)		
Female	77 (61.6%)	48 (38.4%)		
Location			1.709	0.426
Proximal	29 (52.7%)	26 (47.3%)		
Middle	102 (62.6%)	61 (37.4%)		
Distal	133 (61.0%)	85 (39.0%)		
Size			5.695	0.017
<5 cm	167 (65.2%)	89 (34.8%)		
≥5 cm	97 (53.9%)	83 (46.1%)		
Lauren classification			47	<0.01
Intestinal	170 (76.2%)	53 (23.8%)		
Diffuse	94 (44.1%)	119 (55.9%)		
Histology classification			2.656	0.448
Papillary adenocarcinoma	9 (56.2%)	7 (43.8%)		
Tubular adenocarcinoma	192 (58.9%)	134 (41.1%)		
Mucinous adenocarcinoma	21 (72.4%)	8 (27.6%)		
Signet ring cell carcinoma	42 (64.6%)	23 (35.4%)		
Histologic differentiation			3.336	0.343
Well	11 (84.6%)	2 (15.4%)		
Moderately	77 (60.2%)	51 (39.8%)		
Poorly	175 (59.7%)	118 (40.3%)		
Others	1 (50%)	1 (50%)		
Invasion depth			25.748	<0.01
T1	47 (82.5%)	10 (17.5%)		
T2	77 (70.6%)	32 (29.4%)		
T3	129 (52.9%)	115 (47.1%)		
T4	11 (42.3%)	15 (57.7%)		
Lymphatic metastasis			30.767	<0.01
No	128 (83.1%)	38 (16.9%)		
Yes	136 (30.4%)	134 (69.6%)		
Regional lymph nodes			32.746	<0.01
PN0	128 (77.1%)	38 (22.9%)		
PN1	74 (54.4%)	62 (45.6%)		
PN2	45 (45.5%)	54 (54.5%)		
PN3	17 (48.6%)	18 (51.4%)		
Distant metastasis			17.8	<0.01
No	242 (64.5%)	133 (35.5%)		
Yes	22 (36.1%)	39 (63.9%)		
TNM stages			45.868	<0.01
I	74 (82.2%)	16 (17.8%)		
II	75 (72.1%)	29 (27.9%)		
III	90 (52.0%)	83 (48.0%)		
IV	25 (36.2%)	44 (63.8%)		

## Data Availability

All data can be obtained from Li Li, Clinical Research Institute, Zhejiang Provincial People's Hospital, People's Hospital of Hangzhou Medical College, Hangzhou 310014, Zhejiang, China, and No. 158 Shangtang Road, Hangzhou, Zhejiang, 310014, China, Tel: +86-0571-85893486; fax: +86-0571-85131448; and e-mail: lilideshy@163.com.
